# The Identification of Potential Therapeutic Targets for Cutaneous Squamous Cell Carcinoma

**DOI:** 10.1016/j.jid.2019.09.024

**Published:** 2020-06

**Authors:** Angela McHugh, Kenneth Fernandes, Nerime Chinner, Adel F.M. Ibrahim, Amit K. Garg, Garry Boag, Lydia A. Hepburn, Charlotte M. Proby, Irene M. Leigh, Mark K. Saville

**Affiliations:** 1 Division of Cancer Research, School of Medicine, University of Dundee, Dundee, United Kingdom; 2 MRC Protein Phosphorylation and Ubiquitylation Unit, School of Life Sciences, University of Dundee, Dundee, United Kingdom; 3 Division of Molecular and Clinical Medicine, School of Medicine, University of Dundee, Dundee, United Kingdom; 4 Institute of Dentistry, Barts and the London School of Medicine and Dentistry, Queen Mary University of London, London, United Kingdom

**Keywords:** APC/C, anaphase-promoting complex/cyclosome, CRL, cullin-RING ligase, cSCC, cutaneous squamous cell carcinoma, ESCRT, endosomal sorting complexes required for transport, RDEB, recessive dystrophic epidermolysis bullosa, siRNA, small interfering RNA

## Abstract

We performed a small interfering RNA screen to identify targets for cutaneous squamous cell carcinoma (cSCC) therapy in the ubiquitin/ubiquitin-like system. We provide evidence for selective anti-cSCC activity of knockdown of the E3 ubiquitin ligase MARCH4, the ATPase p97/VCP, the deubiquitinating enzyme USP8, the cullin-RING ligase (CRL) 4 substrate receptor CDT2/DTL, and components of the anaphase-promoting complex/cyclosome (APC/C). Specifically attenuating CRL4^CDT2^ by CDT2 knockdown can be more potent in killing cSCC cells than targeting CRLs or CRL4s in general by RBX1 or DDB1 depletion. Suppression of the APC/C or forced APC/C activation by targeting its repressor EMI1 are both potential therapeutic approaches. We observed that cSCC cells can be selectively killed by small-molecule inhibitors of USP8 (DUBs-IN-3/compound 22c) and the NEDD8 E1 activating enzyme/CRLs (MLN4924/pevonedistat). A substantial proportion of cSCC cell lines are very highly MLN4924-sensitive. Pathways that respond to defects in proteostasis are involved in the anti-cSCC activity of p97 suppression. Targeting USP8 can reduce the expression of growth factor receptors that participate in cSCC development. EMI1 and CDT2 depletion can selectively cause DNA re-replication and DNA damage in cSCC cells.

## Introduction

There is a need for improved treatment for cutaneous squamous cell carcinoma (cSCC) in high-risk recessive dystrophic epidermolysis bullosa (RDEB) and immunocompromised patients, including transplant recipients and in the general population (Harwood et al., 2016, Mellerio et al., 2016). This includes better systemically and locally delivered therapy. The cumulative risk of death from cSCC in patients with RDEB is 80% by the age of 55 years, and overall, cSCC causes 25% of skin cancer–related deaths. cSCC, including multiple primary tumors in high-risk individuals, also results in considerable morbidity.

The ubiquitin/ubiquitin-like system plays a widespread role in regulating cellular pathways and processes. It contains multiple classes of proteins including E1 activating enzymes, E2 conjugating enzymes, E3 ligases, receptors for ubiquitin/ubiquitin-like proteins, ATPases, and proteases. Considerable work has been carried out to target this system for cancer therapy, and there are a growing number of small-molecule modulators.

We have shown that proteasome and ubiquitin E1 inhibitors have therapeutic potential for cSCC (McHugh et al., 2018). In this study, we screened using a small interfering RNA (siRNA) library complementary to more than 1,000 genes to identify additional components of the ubiquitin/ubiquitin-like system that could be targeted for cSCC therapy. We assessed the cSCC selectivity compared with that of normal skin cells of knockdown and small-molecule inhibition of targets identified in the screen. We also initiated studies to investigate mechanisms of anti-cSCC activity.

## Results and Discussion

### siRNA screening for potential therapeutic targets

**Figure 1:**
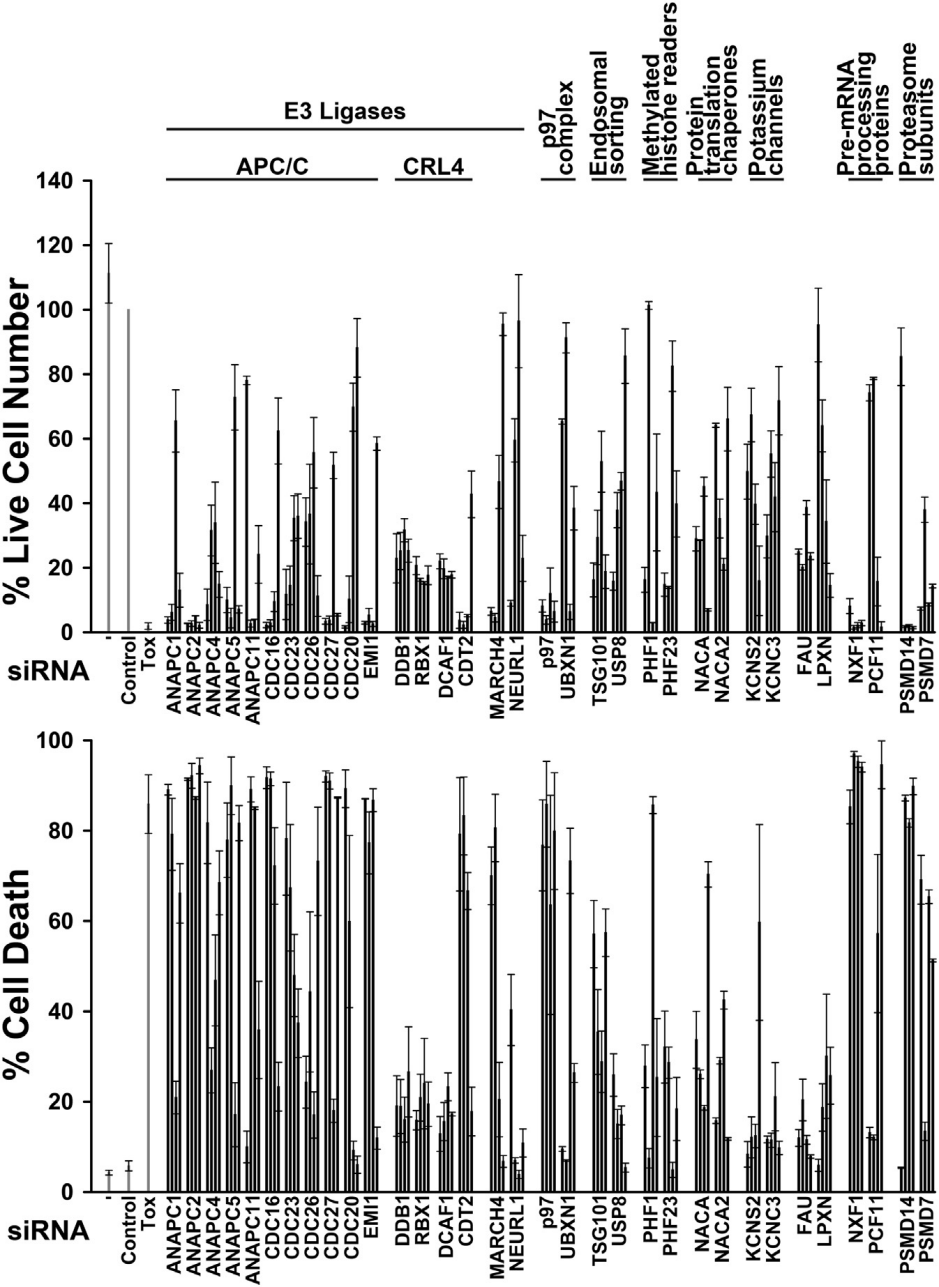
**Targets in the ubiquitin/ubiquitin-like system for which at least two siRNAs substantially reduced viability.** SCCRDEB4 cells were mock transfected (−) or transfected with nontargeting siRNA (Control), cytotoxic siRNA (Tox), or four individual siRNAs for each target. Cell viability (percentage of control live cell number) and the percentage of dead cells were determined by real-time imaging 96 hours after transfection. Values are the mean ± range of two experiments or ± the SD of at least three experiments. The cellular roles of targets are indicated. APC/C, anaphase-promoting complex/cyclosome; CRL, cullin-RING ligase; SD, standard deviation; siRNA, small interfering RNA.

**Supplementary Figure S1:**
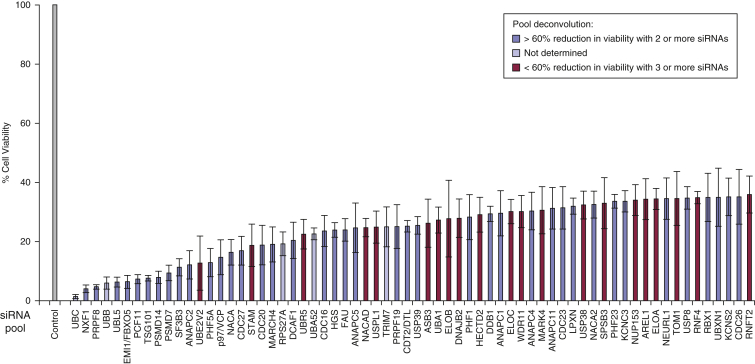
**Sixty-six siRNA pools in the ubiquitin/ubiquitin-like pathway library reduced viability by more than 65%.** SCCRDEB4 cells were transfected with a pool of four nontargeting siRNAs (Control) or pools of four siRNAs complementary to 1,186 ubiquitin/ubiquitin-like system–related genes. Cell viability was determined 96 hours after transfection by ATPase assay. The average Z-prime for the screen was 0.82. Values are the mean percentage of control ± SD of three determinations. The effects of four individual siRNAs are indicated (Pool deconvolution). siRNA, small interfering RNA.

**Supplementary Figure S2:**
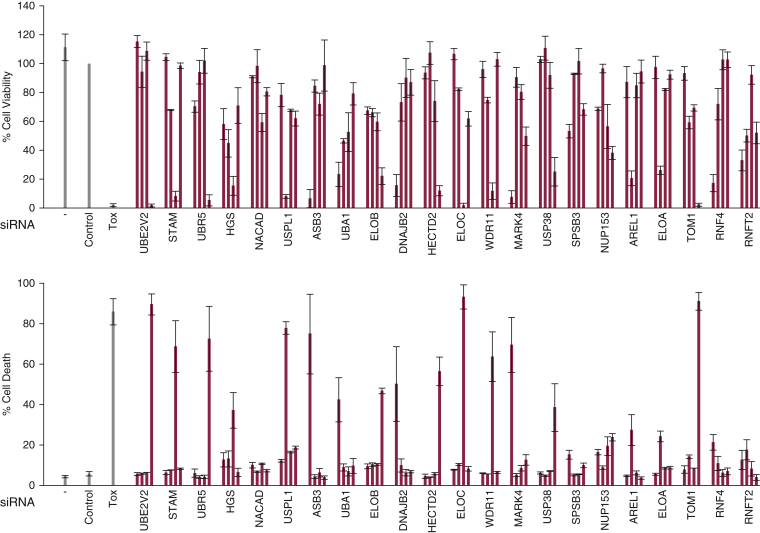
**Targets from the primary screen for which multiple siRNAs did not cause a substantial reduction in viability.** SCCRDEB4 cells were transfected with four individual siRNAs per target gene and cell viability (percentage of control live cell number) and the percentage of dead cells were assayed by real-time imaging. Values are the mean ± range of two determinations. For many of these targets, only one siRNA had a substantial effect on viability and death. Although it remains possible this is due to greater efficacy of target knockdown by the siRNA with the strongest phenotype, it could also result from off-target effects. siRNA, small interfering RNA; Tox, cytotoxic small interfering RNA.

A cell line derived from a primary RDEB cSCC (SCCRDEB4) was transfected with pools of four siRNAs targeting 1,186 ubiquitin/ubiquitin-like pathway-linked genes (MacKay et al., 2014). Cell viability was reduced by >65% by siRNA pools targeting 66 genes (Supplementary Figure S1). Of these, six encoded for ubiquitin system–related components of the spliceosome, which we have investigated in detail (Hepburn et al., 2018). To identify genuine targets, we determined the effects on viability and death in SCCRDEB4 cells of four individual siRNAs. Variations in the responses to siRNAs could arise from differences in their effectiveness in knocking down the target and its splice variants as well as false-negative or false-positive off-target effects. At least two siRNAs reduced cell viability (live cell number) by >60% for 34 genes and increased cell death to >30% for 22 genes (Figure 1). Some of these genes encoded for proteins that are subunits of the same complexes, others for proteins that participate in the same cellular processes. For most of the remaining targets, only a single siRNA had a robust effect on cell viability and death (Supplementary Figure S2).

### Additional target validation

To further investigate the therapeutic potential of suppression of genes for which multiple individual siRNAs had a phenotype, we determined the effects of the siRNAs on viability and death in normal skin cells (fibroblasts and keratinocytes) and cell lines derived from metastasis in an RDEB (SCCRDEBMet) and a transplant patient (SCCTMet). A cytotoxic siRNA was used in each experiment as a control for transfection efficiency. In addition, we determined the extent of target protein knockdown in normal and cSCC cells. The effects of small-molecule inhibitors on viability and death in normal skin cells and cSCC cell lines were also evaluated.

#### MARCH4

MARCH4 is a little-studied transmembrane E3 ubiquitin ligase that is localized to the Golgi apparatus (Bartee et al., 2004, Bauer et al., 2017, Samji et al., 2014). Ectopically expressed MARCH4 increases lysosomal degradation of several proteins that promote immune responses, and its knockdown increases cell surface levels of the scaffolding protein tetraspanin CD81 (Bartee et al., 2010). However, there are likely to be additional MARCH4 substrates (Nathan and Lehner, 2009).

**Figure 2:**
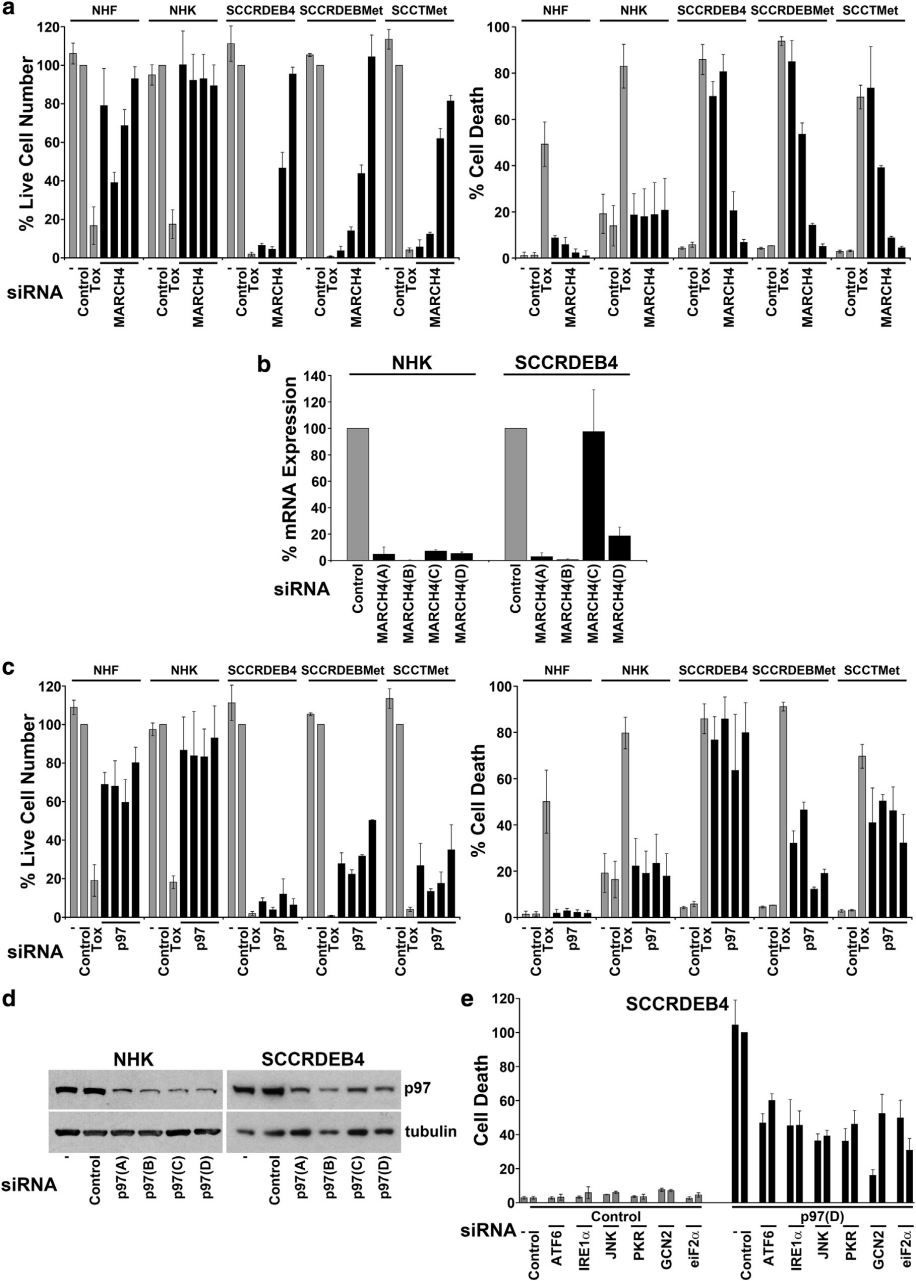
**MARCH4 and p97 knockdown selectively kills cSCC cells.** Normal skin cells (NHF and NHK) and cSCC lines (SCCRDEB4, SCCRDEBMet, and SCCTMet) were mock transfected (−) or transfected with siRNAs as indicated. (**a, c**) Cell viability and the percentage of dead cells were determined by real-time imaging following transfection with four siRNAs targeting (a) MARCH4 or (c) p97: mean ± SD of at least three experiments (NHK, NHF, and SCCRDEB4 cells) or ± the range of two experiments (SCCRDEBMet and SCCTMet cells). (**b**) *MARCH4* mRNA knockdown: mean ± range of two experiments. (**d**) p97 protein knockdown. (**e**) Co-transfection of control or p97(D) siRNAs with siRNAs targeting genes involved in responding to defects in proteostasis (two siRNAs per target): mean percentage of cell death in p97(D) and control siRNA-transfected cells ± SD of four experiments. cSCC, cutaneous squamous cell carcinoma; NHF, normal human fibroblast; NHK, normal human keratinocyte; SD, standard deviation; siRNA, small interfering RNA; Tox, cytotoxic small interfering RNA.

*MARCH4* siRNAs had little effect on death in normal skin cells, whereas two *MARCH4* siRNAs caused a reduction in viability and increased death in cSCC cell lines (Figure 2a). We were unable to detect MARCH4 protein with available antibodies (data not shown). However, we confirmed that *MARCH4* mRNA levels were reduced in normal human keratinocytes by *MARCH4* siRNAs and that in SCCRDEB4 cells, the siRNAs most potent in killing cSCC cells caused the largest reduction in *MARCH4* mRNA levels (Figure 2b).

#### p97/VCP

p97 is an ATPase that unfolds ubiquitinated proteins and extracts them from membranes, cellular structures, and complexes (van den Boom and Meyer, 2018, Ye et al., 2017). Through this, p97 can facilitate substrate degradation by the proteasome, and it can also regulate substrate activity, complex assembly, and membrane fusion. p97 participates in a wide range of cellular processes. It maintains protein homeostasis (proteostasis) by promoting the proteasomal degradation of misfolded proteins associated with the endoplasmic reticulum, ribosomes, and mitochondria. It also regulates lysosomes and autophagosome maturation. Other roles of p97 include the control of key proteins involved in signal transduction, DNA replication, and DNA repair. Distinct p97 complexes are involved in particular cellular processes; p97 associates with numerous adaptors and cofactors that recruit substrates and participate in substrate processing (Stach and Freemont, 2017, Ye et al., 2017).

*p97* siRNAs killed cSCC lines but not normal skin cells, whereas p97 was depleted in both normal human keratinocytes and SCCRDEB4 cells (Figure 2c and d). We investigated whether p97 knockdown–induced death was dependent on pathways that sense defects in proteostasis. Death due to depletion of p97 was attenuated by suppression of proteins involved in responses to the accumulation of unfolded proteins in the endoplasmic reticulum (ATF6, IRE1a/JNK1, and PKR/eiF2α) and to amino acid depletion (GCN2/eiF2α) (Figure 2e) (McConkey, 2017, Parzych et al., 2015). cSCCs have frequent gene copy number changes, and UV-induced cSCCs in particular have extremely high gene mutation rates (Cho et al., 2018, Inman et al., 2018, South et al., 2014). These alterations can confer greater dependency on mechanisms of proteostasis by causing imbalanced protein production, which can generate free components of complexes that cannot fold appropriately, and through the generation of proteins that are misfolded because of mutations (Deshaies, 2014, Vekaria et al., 2016). Consistent with greater basal proteotoxic stress, there is an increase in the expression of proteasome subunits and Ser51 phosphorylated eiF2α in cSCC cell lines compared with normal skin cells (McHugh et al., 2018).

**Supplementary Figure S3:**
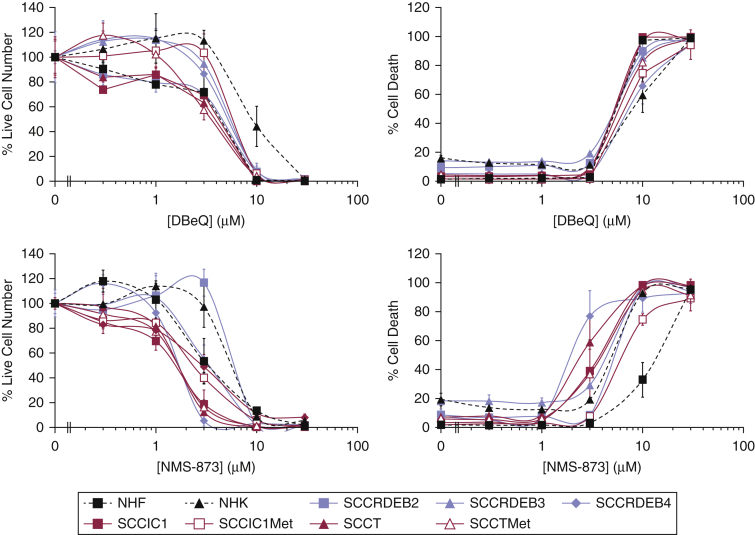
**The p97 inhibitors DBeQ and NMS-873 do not display a high level of selectivity for cSCC cells.** Normal skin cells (NHF and NHK) and cSCC cell lines derived from RDEB (SCCRDEB), immunocompetent (SCCIC), and immunocompromised transplant (SCCT) patients were treated for 72 hours with p97 inhibitors. Cell viability (percentage of control live cell number) and the percentage of dead cells were assayed by real-time imaging. cSCC, cutaneous squamous cell carcinoma; NHF, normal human fibroblast; NHK, normal human keratinocyte; RDEB, recessive dystrophic epidermolysis bullosa.

Numerous small-molecule p97 inhibitors have been developed (Chapman et al., 2015, Vekaria et al., 2016, Ye et al., 2017). The well-characterized p97 inhibitors DBeQ and NMS-873 were at best modestly selective for effects on viability and death in cSCC lines compared with normal skin cells, and the sensitivity of the most responsive cSCC lines was around average for tumor-derived cells (Supplementary Figure S3) (Magnaghi et al., 2013, Parzych et al., 2015). It is possible that the differences in the cSCC selectivity of these inhibitors and p97 knockdown are due to divergent effects on the spectrum of p97-regulated pathways. For example, the effects of p97 knockdown could be influenced by competition of binding partners for residual p97. The potency of p97 inhibitors can be differentially affected by p97-interacting proteins, potentially resulting in preferential effects of inhibitors on particular complexes (Gui et al., 2016). It would be of interest to compare additional p97 inhibitors to determine if greater cSCC selectivity can be achieved.

#### USP8

Sorting of endocytosed, activated cell surface receptors for lysosomal degradation or recycling to the plasma membrane is mediated by endosomal sorting complexes required for transport (ESCRT). Ubiquitination of receptors promotes ESCRT-mediated lysosomal trafficking. USP8/UBPY-mediated deubiquitination of some ESCRT-associated receptors can facilitate their recycling, and USP8 also regulates endocytic sorting by stabilizing ESCRT-0 proteins HGS, STAM, and STAM2 (D'Arcy et al., 2015, Niendorf et al., 2007, Wright et al., 2011).

**Figure 3:**
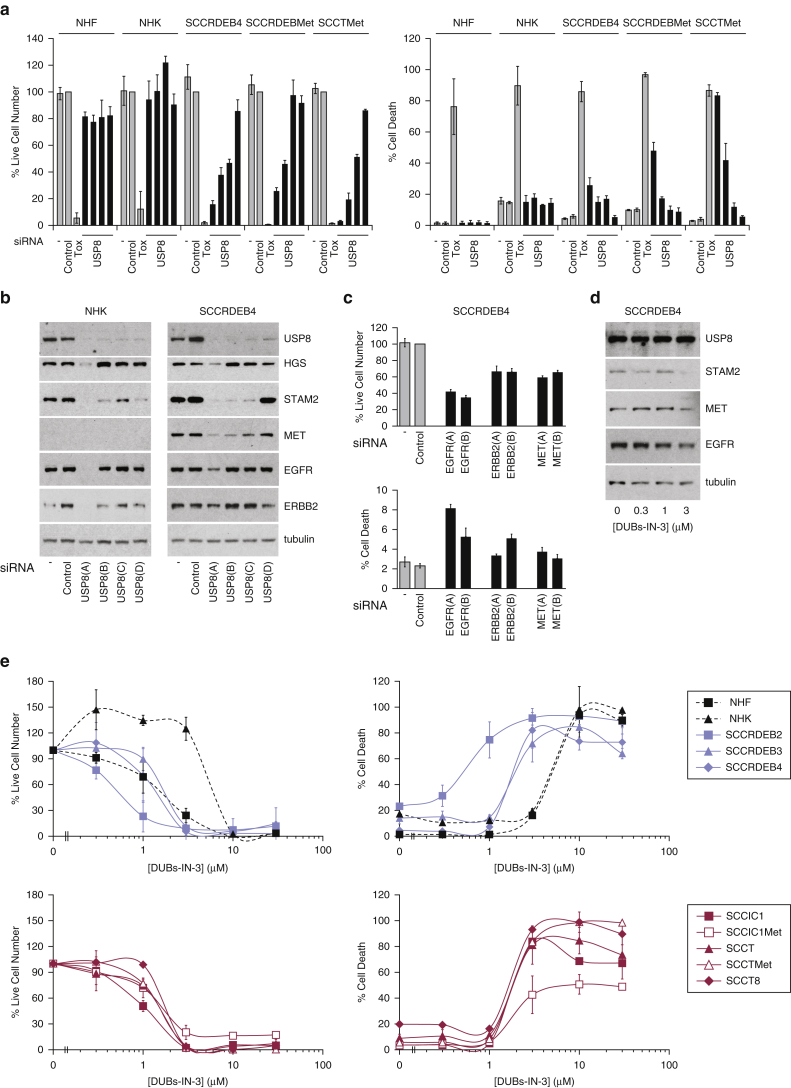
**Suppression of USP8 has selective anti-cSCC activity.** (**a**) Cell viability and the percentage of dead cells were determined by real-time imaging 96 hours after transfection with USP8 siRNAs: mean ± SD of at least three experiments (NHK, NHF, and SCCRDEB4 cells) or ± the range of two experiments (SCCRDEBMet and SCCTMet cells). (**b**) Protein expression was analyzed by western blotting 48 hours after transfection with USP8 siRNAs. (**c**) Cell viability and the percentage of dead cells 96 hours after transfection with two siRNAs targeting growth factor receptors: mean ± SD of four experiments. (**d**) Western blot analysis of SCCRDEB4 cells treated with the USP8 inhibitor DUBs-IN-3 for 24 hours. (**e**) Cell viability and the percentage of dead cells 72 hours after initiation of DUBs-IN-3 treatment: mean ± range of two experiments. cSCC, cutaneous squamous cell carcinoma; NHF, normal human fibroblast; NHK, normal human keratinocyte; SD, standard deviation; siRNA, small interfering RNA; Tox, cytotoxic small interfering RNA.

*USP8* siRNAs reduced viability and increased death in cSCC lines but had little effect in normal skin cells (Figure 3a). The anti-cSCC potency of these siRNAs reflected their ability to reduce USP8 protein levels (Figure 3b). siRNA *USP8(A)* depleted USP8 to the greatest extent, and this was associated with reduced expression of growth factor receptors MET, EGFR, and ERBB2, along with HGS and STAM2. siRNAs *USP8(B)* and *(C)* reduced expression of MET and STAM2. This is consistent with a role of USP8 in protecting these proteins from degradation.

Small-molecule USP8 inhibitors have been identified including DUBs-IN-3/compound 22c (Colombo et al., 2010, D'Arcy et al., 2015). DUBs-IN-3 killed cSCC lines at lower concentrations than normal skin cells (Figure 3e). Reduced MET, EGFR, and STAM2 expression was consistent with attenuation of USP8 at DUBs-IN-3 concentrations that selectively increased death in cSCC cells (Figure 3d). We observed that directly targeting MET, EGFR, or ERBB2 was sufficient to impact cSCC cell viability (Figure 3c). MET and EGFR/ERBB2 signaling pathways can contribute to driving cSCC development, and a subset of cSCCs respond to EGFR inhibitors (Cataisson et al., 2016, Harwood et al., 2016, Mellerio et al., 2016). Suppression of USP8 could provide a means to simultaneously interfere with multiple therapeutically relevant receptors, which could overcome resistance because of receptor redundancy or cross-talk.

#### Anaphase-promoting complex/cyclosome (APC/C)

The APC/C is a multisubunit E3 ligase that coordinates transitions through the cell cycle by targeting key proteins for proteasomal degradation (Alfieri et al., 2017, Zhou et al., 2016). CDC20 and CDH1/FZR1 are coactivators that associate with the core APC/C complex at different stages of the cell cycle and recruit overlapping but distinct sets of substrates for ubiquitination. APC/C^CDC20^ drives progression through mitosis (Kapanidou et al., 2017). APC/C^CDC20^ is inhibited by the spindle assembly checkpoint until chromosomes are properly attached to spindle microtubules. Following transition through the spindle assembly checkpoint, APC/C^CDC20^ promotes the degradation of securin and cyclin B, allowing chromatid segregation and mitotic exit. In late mitosis and G1, APC/C^CDH1^ participates in promoting licensing of DNA for replication by allowing the recruitment of the prereplicative complex to replication origins (Hernández-Carralero and Cabrera, 2018, Moreno and Gambus, 2015). This involves APC/C^CDH1^-mediated degradation of geminin, which is an inhibitor of the key replication licensing factor CDT1, and suppression of cyclin A and B CDK complexes, which inhibit replication licensing. The attenuation of cyclin/CDKs also contributes to APC/C^CDH1^-mediated blockade of cell-cycle progression. In late G1, S, and G2 phase, cells’ APC/C^CDH1^ activity is inhibited in part by E2F-dependent induction of the APC/C-binding protein EMI1. This prevents further rounds of DNA replication licensing and permits entry into S phase (Abbas and Dutta, 2017, Cappell et al., 2016, Reimann et al., 2001). APC/C^CDH1^ thus participates in allowing licensing to occur only before the onset of DNA replication, which contributes to ensuring that the genome is just duplicated once per cell cycle.

**Figure 4:**
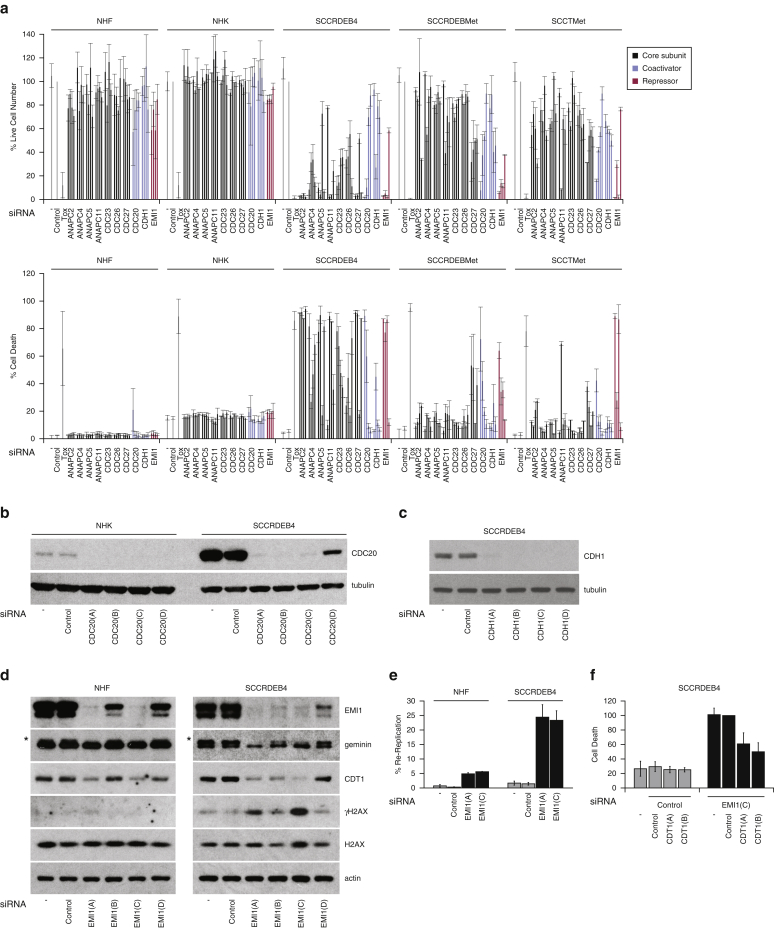
**APC/C suppression and derepression have potential for cSCC therapy**. (**a**) Cells were transfected with four siRNAs per target. Cell viability and the percentage of dead cells were assessed 96 hours after transfection by real-time imaging: mean ± SD of at least three experiments (CDC20, CDH1, and EMI1 in NHK, NHF, and SCCRDEB4 cells) or ± range of two experiments. (**b–d**) Protein expression analyzed 48 hours after transfection with siRNAs targeting (b) CDC20, (c) CDH1, and (d) EMI1. Geminin (upper band _*_) and CDT1 expression was reduced by EMI1 knockdown. (**e**) The percentage of cells in which DNA was re-replicated 72 hours after siRNA transfection: mean ± range of two experiments. (**f**) Cell death 72 hours after siRNA co-transfection: percentage of that in EMI1(C) and control siRNA-transfected cells: mean ± SD of three experiments. APC/C, anaphase-promoting complex/cyclosome; cSCC, cutaneous squamous cell carcinoma; NHF, normal human fibroblast; NHK, normal human keratinocyte; SD, standard deviation; siRNA, small interfering RNA; Tox, cytotoxic small interfering RNA.

siRNA pools targeting 10 of the 14 core subunits of the APC/C substantially reduced viability in our primary screen, and multiple individual APC/C core subunit siRNAs killed SCCRDEB4 cells (Supplementary Figure S1 and Figure 4a). There was little effect on viability or death of targeting core APC/C subunits in normal skin cells, but effects on SCCRDEBMet and SCCTMet cells were also generally modest (Figure 4a). Interfering with coactivators CDC20 and CDH1 provides a means to specifically suppress different APC/C functions. Two of the *CDC20* siRNAs selectively killed cSCC cell lines, whereas they robustly reduced CDC20 protein expression in both normal skin cells and SCCRDEB4 cells (Figure 4b). siRNA *CDC20(D)* was the least effective in depleting CDC20 and had no effect on viability or death. Three *CDH1* siRNAs depleted CDH1 without causing a high level of death in cSCC cells (Figure 4a and c). These data suggest that CDC20 rather than CDH1 has potential as a therapeutic target for cSCC.

**Supplementary Figure S4:**
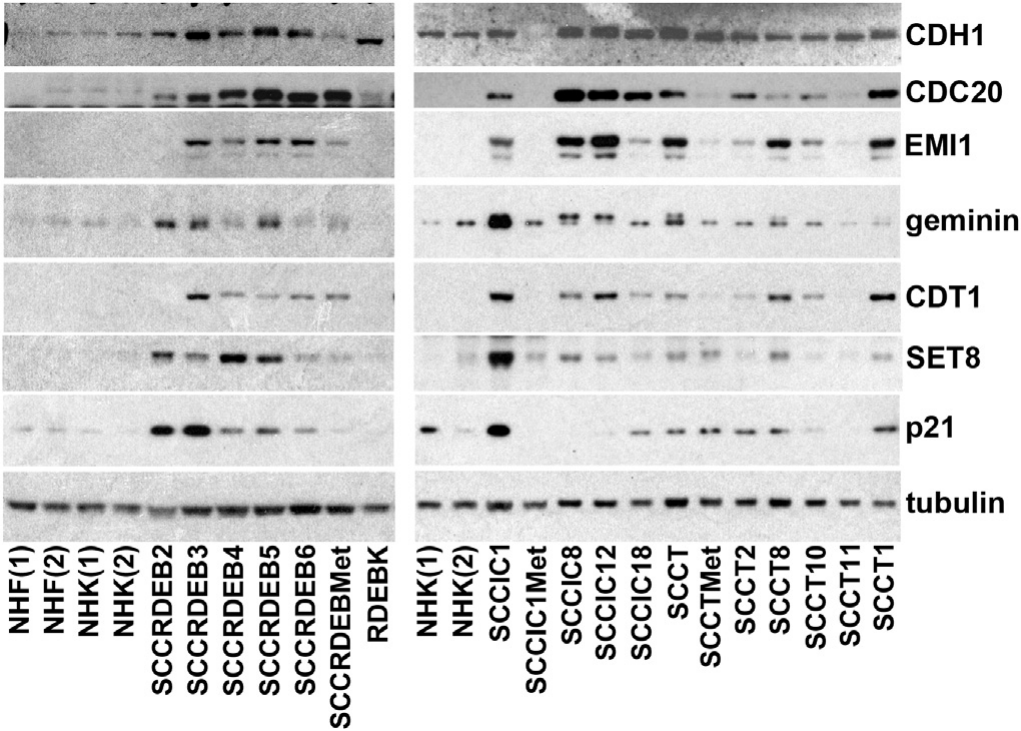
**The APC/C coactivators CDH1 and CDC20, the APC/C repressor EMI1, and proteins that promote DNA replication licensing are frequently upregulated in cSCC lines.** Protein expression was analyzed by western blotting in normal skin cells (NHF, NHK, and RDEBK) and a panel of lines derived from primary and metastatic cSCCs in RDEB (SCCRDEB), immunocompetent (SCCIC), and transplant (SCCT) patients. NHF1 and 2 and NHK1 and 2 were from different donors. APC/C, anaphase-promoting complex/cyclosome; cSCC, cutaneous squamous cell carcinoma; NHF, normal human fibroblast; NHK, normal human keratinocyte; RDEB, recessive dystrophic epidermolysis bullosa.

CDC20 is essential for mitosis, but a high level of suppression is required to block cell-cycle progression in normal cells and in many tumor cells (Baumgarten et al., 2009, Crawford et al., 2016, Jin et al., 2010, Kidokoro et al., 2008, Li et al., 2014, Li et al., 2007, Taniguchi et al., 2008, Wirth et al., 2004, Zhang et al., 2014). The robust effects of targeting APC/C core subunits and CDC20 in SCCRDEB4 cells likely reflects sensitivity to partial suppression of APC/C. A need to maintain elevated levels of CDC20 for survival may contribute to cell death induced by CDC20 depletion in SCCRDEB4 and SCCRDEBMet cells (Supplementary Figure S4). Small-molecule antagonists of CDC20/CDH1 (TAME) and CDC20 (apcin) are at an early stage of development (Kapanidou et al., 2017, Sackton et al., 2014, Wang et al., 2015, Zeng et al., 2010). Antimitotic agents, including compounds that interfere with microtubule dynamics and Aurora A and PLK-1 inhibitors, act in part by attenuating APC/C^CDC20^ by activating the spindle assembly checkpoint (Olziersky and Labidi-Galy, 2017). Previous studies indicate PLK-1 is a potential target for cSCC therapy (Watt et al., 2011). Direct APC/C^CDC20^ suppression may be a better therapeutic approach than spindle assembly checkpoint activation because directly targeting APC/C^CDC20^ attenuates premature exit from mitosis (Huang et al., 2009).

We also observed that siRNAs targeting the APC/C repressor EMI1 killed cSCC lines with no effect on death in normal skin cells (Figure 4a). This included SCCRDEBMet and SCCTMet cells, which were relatively resistant to suppression of APC/C. The anti-cSCC potency of the siRNAs was consistent with the extent of EMI1 knockdown (Figure 4d). We investigated the role of DNA re-replication in EMI1 knockdown-dependent death. EMI1 depletion caused a greater increase in DNA re-replication (greater than G2/M DNA content) in cSCC cells than in normal human fibroblasts along with a tumor cell–selective increase in the DNA damage marker γH2AX (Figure 4d and e and Supplementary Figure S5a). Furthermore, death induced by EMI1 depletion was dependent on the DNA replication licensing factor CDT1 (Figure 4f). This is in line with previous studies in other cell types showing that reduced viability following EMI1 suppression can be caused by APC/C^CDH1^-dependent DNA re-replication leading to DNA damage (Machida and Dutta, 2007, Neelsen et al., 2013, Shimizu et al., 2013, Verschuren et al., 2007).

High expression of replication licensing factors increases susceptibility to re-replication (Benamar et al., 2016, Muñoz et al., 2017, Vaziri et al., 2003). Elevated expression of replicating licensing proteins including CDT1 compared with normal skin cells consequently provides a mechanism that could contribute to increased re-replication in cSCC cells on targeting components of the protective machinery including EMI1 (Supplementary Figure S4). This is supported by the observation that depletion of CDT1 diminished EMI1 knockdown–induced death (Figure 4f). EMI1 increases expression of the CDT1 repressor geminin by attenuating APC/C^CDH1^-mediated geminin degradation. In normal cells, geminin accumulates inactive CDT1 in preparation for the next round of replication licensing (Ballabeni et al., 2004, Ballabeni et al., 2013). EMI1 protein expression was strongly upregulated in cSCC cell lines, which could prime cSCC cells for re-replication by contributing to the accumulation of CDT1 (Supplementary Figure S4). Consistent with this, we observed that EMI1 and CDT1 protein expression was frequently co-upregulated in cSCC cell lines, and EMI1 knockdown reduced both geminin and CDT1 protein levels (Supplementary Figure S4 and Figure 4d).

#### Cullin-RING Ligase (CRL) 4^CDT2^

**Figure 5:**
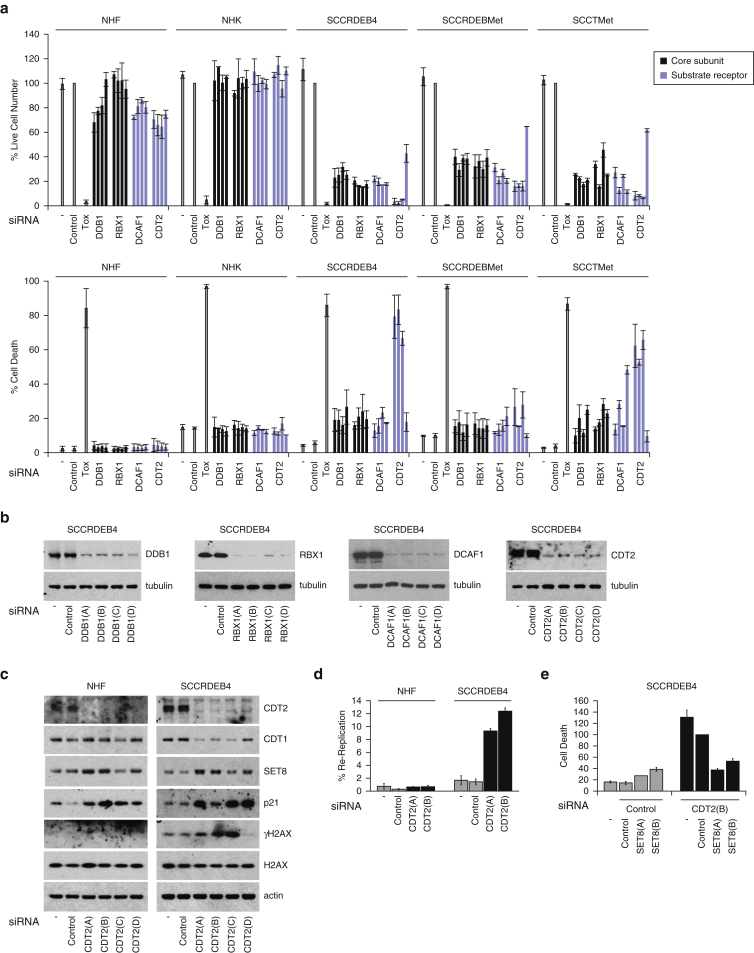
**CRL4^CDT2^ is a potential therapeutic target for cSCC.** (**a**) Cells were transfected with four siRNAs targeting the CRL4 adaptor DDB1 and substrate receptors DCAF1 and CDT2 and the CRL/CRL4 RING finger protein RBX1. Viability and the percentage of dead cells were assessed by real-time imaging after 96 hours: mean ± range of two experiments. (**b, c**) Protein expression was analyzed after 48 hours with four siRNAs targeting (b) DDB1, RBX1, DCAF1, and CDT2 in SCCRDEB4 cells and (c) CDT2 in NHF and SCCRDEB4 cells. (**d**) The percentage of cells in which DNA was re-replicated 72 hours after siRNA transfection: mean ± range of two experiments. (**e**) Viability and death were assessed by real-time imaging 96 hours after siRNA co-transfection: percentage of cell death in CDT2(B) and control siRNA transfected cells: mean ± SD of three experiments. CRL, cullin-RING ligase; cSCC, cutaneous squamous cell carcinoma; NHF, normal human fibroblast; NHK, normal human keratinocyte; SD, standard deviation; siRNA, small interfering RNA; Tox, cytotoxic small interfering RNA.

CRLs are a very large family of multisubunit E3 ubiquitin ligases containing 1 of 8 cullin scaffolds (Bulatov and Ciulli, 2015, Jang et al., 2018). CRL4s are composed of cullin4(A/B), DDB1 adaptor, RBX1 E2-binding RING finger, and one of many substrate-recruiting receptors (Hannah and Zhou, 2015). RBX1 is a component of multiple CRLs, cullin4 and DDB1 are present in all CRL4s, and the receptors determine substrate specificity and consequently the cellular roles of CRL4 complexes. siRNAs targeting DDB1, RBX1, and the CRL4 substrate receptors DCAF1/VPRBP and CDT2/DTL reduced viability in cSCC cell lines but had only a modest effect in normal skin cells (Figure 5a). This indicates targeting CRL4s, in particular CRL4^DCAF1^ or CRL4^CDT2^, can have selective anti-cSCC activity. In support of this, suppression of CRL4s protects against UV-induced skin cancer in mice (Hannah and Zhou, 2013). CDT2 knockdown could cause more death in cSCC cells than targeting RBX1 or DDB1 (Figure 5a and b). RBX1/DDB1 depletion may elicit prosurvival responses through attenuation of CRLs in addition to CRL4^CDT2^. Similar to EMI1, CRL4^CDT2^ is involved in protecting against DNA re-replication (Abbas and Dutta, 2011, Abbas and Dutta, 2017, Moreno and Gambus, 2015). During S-phase and DNA repair, CRL4^CDT2^ promotes the DNA replication–coupled proteasomal degradation of PCNA–bound proteins involved in promoting replication licensing including CDT1, SET8, and p21 (Havens and Walter, 2011, Hernández-Carralero and Cabrera, 2018, Scrima et al., 2011). CDT2 was efficiently knocked down in both normal human fibroblasts and cSCC cells (Figure 5c). This resulted in the accumulation of the CRL4^CDT2^ substrates SET8 and p21 but not CDT1. Failure to accumulate CDT1 is consistent with previous observations in other cell types and can result from context-dependent CDT1 regulatory mechanisms (Benamar et al., 2016). We observed that CDT2 depletion caused cSCC-selective DNA re-replication and DNA damage and that CDT2 knockdown–induced death in cSCC cells was SET8-dependent (Figure 5c–e and Supplementary Figure S5a). This is consistent with a key role of re-replication in the antitumor activity of CDT2 suppression (Benamar et al., 2016, Olivero et al., 2014).

**Figure 6:**
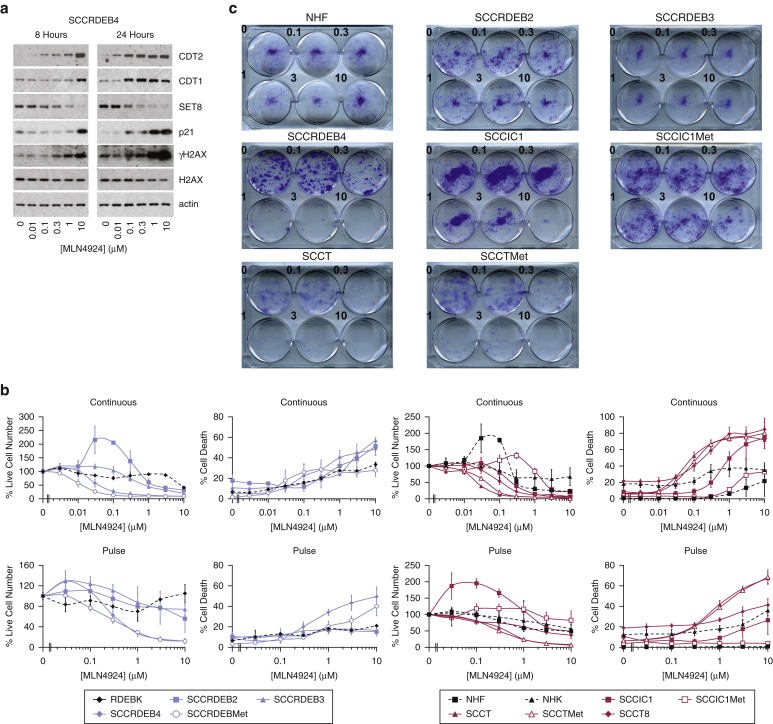
**A subset of cSCC lines are more sensitive than normal skin cells to death induced by MLN4924.** (**a**) SCCRDEB4 cells were incubated with the NEDD8 E1 activating enzyme/CRL inhibitor MLN4924 for 8 and 24 hours. Protein expression was analyzed by western blotting. (**b**) Normal skin cells (NHF, NHK, and RDEBK) or cSCC lines were continuously incubated with MLN4924 for 72 hours (Continuous) or treated with MLN4924 for 8 hours and then maintained in drug-free medium for a further 64 hours (Pulse). Cell viability and death were assessed by real-time imaging: mean ± SD of three experiments. (**c**) Cells were incubated with the indicted concentration of MLN4924 (μM) for 8 hours (comparable to the pulse) and then maintained in drug-free medium to allow colony formation. cSCC, cutaneous squamous cell carcinoma; CRL, cullin-RING ligase; NHF, normal human fibroblast; NHK, normal human keratinocyte; SD, standard deviation.

**Supplementary Figure S6:**
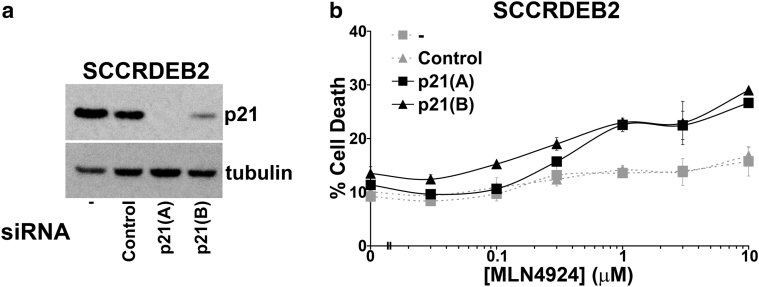
**p21 knockdown partially overcomes resistance to MLN4924.** SCCRDEB2 cells that are relatively insensitive to MLN4924 were transfected with p21 siRNAs. (**a**) p21 protein knockdown assessed by western blotting 48 hours after transfection. (**b**) 48 hours after transfection SCCRDEB2 cells were exposed to an 8-hour pulse of MLN4924. Cell death was determined by real-time imaging 72 hours after initiating treatment with the inhibitor. p21 knockdown increased the level of cell death caused by an 8-hour pulse of MLN4924. cSCC, cutaneous squamous cell carcinoma; siRNA, small interfering RNA.

There are no direct small-molecule inhibitors of CDT2. However, MLN4924/pevonedistat, an inhibitor of the NEDD8-activating enzyme, attenuates CRLs by blocking their NEDDylation (Abidi and Xirodimas, 2015). DNA re-replication due at least in part to CRL4^CDT2^ suppression can make a substantial contribution to the antitumor activity of this inhibitor, although interference with other pathways is also involved (Abbas and Dutta, 2017, Benamar et al., 2016, Zhou et al., 2018). In cSCC cells, MLN4924 caused DNA re-replication and altered expression of proteins involved in promoting replication licensing, although the pattern of changes was different from that caused by CDT2 knockdown (Figures 6a and 5c and Supplementary Figure S5b). It also increased γH2AX, indicative of DNA damage. Viability was reduced at low MLN4924 concentrations in SCCRDEB4, SCCRDEBMet, SCCT, and SCCTMet cells treated continuously for 72 hours (Figure 6b). Comparison with the GDSC Database (Release 8.0) indicates these cSCC lines are in the very highly MLN4924/pevonedistat-sensitive subset of cancer-derived cells (Yang et al., 2013). Death in SCCIC1, SCCT, SCCTMet, and SCCT8 cells was more sensitive than in normal skin cells to continuous MLN4924 treatment. In addition, SCCRDEB4, SCCT, and SCCTMet cells were selectively killed by an 8-hour pulse of MLN4924, which mimics systemically delivered inhibitor pharmacokinetics (Swords et al., 2015). Clonogenic assays confirmed these differences in sensitivity to a pulse of MLN4924 (Figure 6c). At low concentrations, MLN4924 promoted growth in SCCRDEB2 and SCCIC1 cells. Enhanced cell growth has been observed previously but is unusual and requires additional investigation (McMillin et al., 2012). p21 expression was high in three of the cSCC lines most insensitive to a pulse of MLN4924 (SCCIC1, SCCRDEB2, and SCCRDEB3) and p21 knockdown enhanced MLN4924-induced cell death in SCCRDEB2 cells (Supplementary Figures S4 and S6). This is consistent with previous reports that p21 can be protective against MLN4924 (Blank et al., 2013, Lin et al., 2010). Elevated basal p21 may consequently be a marker for resistance of cSCC cells to MLN4924. Wild-type p53 can protect against MLN4924 in part through p21 induction; however, p53 is mutated in all cSCC lines (Lin et al., 2010, Malhab et al., 2016). Given the particular efficacy of CDT2 knockdown in killing cSCC cells, the differences in modulation of CRL4^CDT2^ substrates observed with CDT2 depletion and MLN4924, and the effects of MLN4924 on multiple pathways, it would be of interest to develop CDT2-specific inhibitors.

## Materials and Methods

### Cell culture

Cells were maintained and plated as previously described (McHugh et al., 2018). SCCRDEBMet (SCCRDEB70) and RDEBK cells were provided by Andrew P. South (Thomas Jefferson University), Jemima E. Mellerio (King's College London), and Julio C. Salas-Alanís (DEBRA Mexico). SCCT (previously published as MET1), SCCTMet (previously published as MET4), SCCIC1, and SCCIC1Met cell lines are derived from paired primary tumors and metastases (Hassan et al., 2019, Proby et al., 2000, Watt et al., 2011).

### siRNA transfection

**Supplementary Figure S7:**
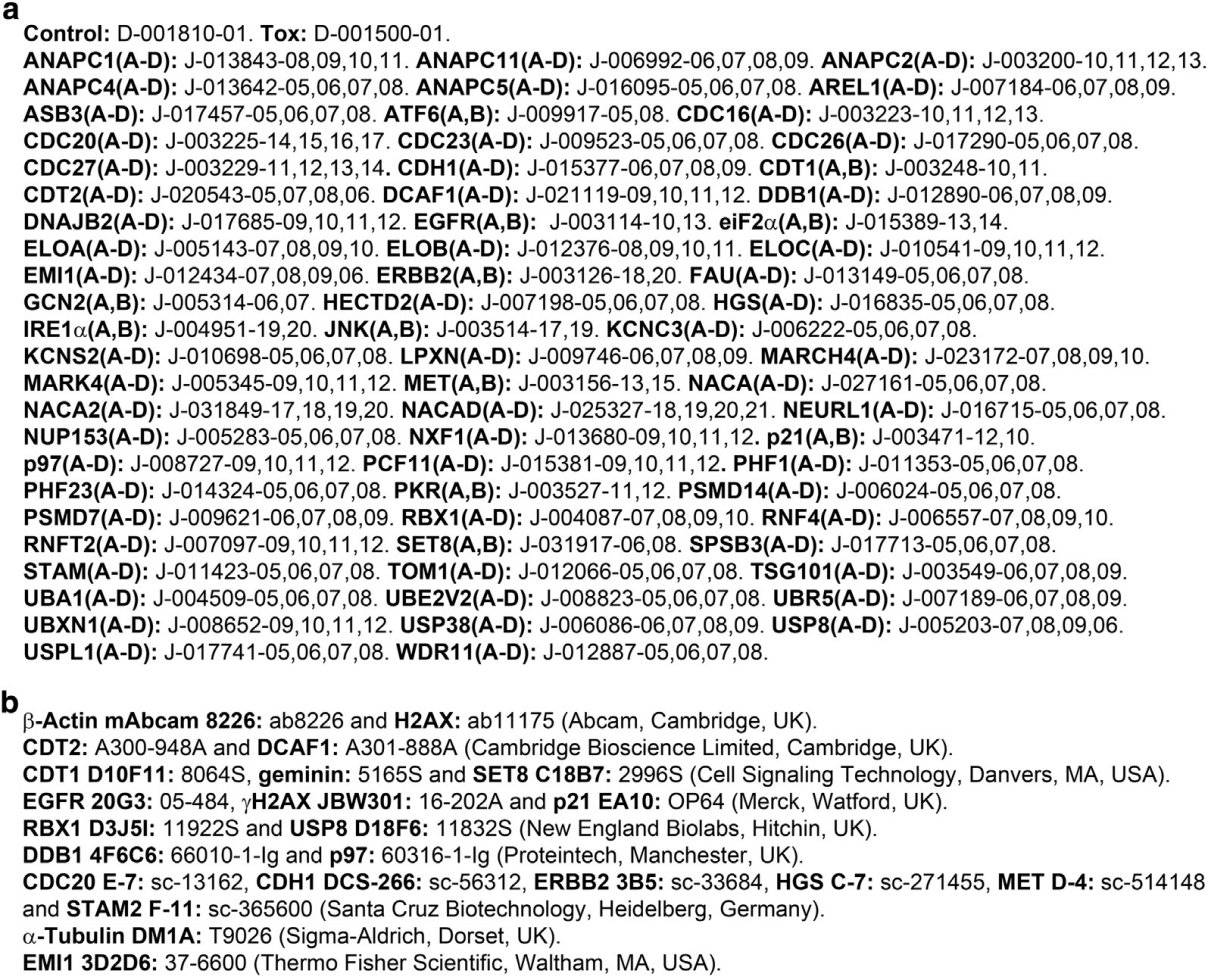
**Materials used in this study.** (**a**) siRNAs. (**b**) Primary antibodies. siRNA, small interfering RNA.

Dharmacon ON-TARGETplus modified siRNAs (Thermo Fisher Scientific, Waltham, MA) were used in this study to minimize off-target effects. Reverse transfection with synthetic siRNA duplexes (10 nM) was performed using Invitrogen Lipofectamine RNAiMAX (Thermo Fisher Scientific). The library used for the primary screen containing pools of four siRNAs per gene was detailed previously (MacKay et al., 2014). Additional siRNAs are listed in Supplementary Figure S7a.

### Inhibitor treatment

Inhibitors used in this work were DBeQ and NMS-873 (Selleckchem, Houston, TX), DUBs-IN-3/compound 22c (Medchemexpress, South Brunswick Township, NJ), and MLN4924 (Boston Biochem, Cambridge, MA).

### Cell viability assays

For the primary screen, viability was measured by ATPase assay 96 hours after siRNA transfection (MacKay et al., 2014). Where indicated, live cell number and cell death were analyzed 96 hours after siRNA transfection or 72 hours after the initiation of inhibitor treatment using an Incucyte ZOOM real-time imager (Essen BioScience Ltd, Welwyn Garden City, United Kingdom) and the CellTox Green Cytotoxicity Assay (Promega, Madison, WI). For clonogenic assays, MLN4924 was added for 8 hours, and cells were maintained in drug-free medium for up to 2 weeks. Colonies were fixed in 10% methanol and 10% acetic acid and stained with crystal violet.

### DNA re-replication

To assess re-replication (>G2/M DNA content), fixed DAPI-stained samples were analyzed for DNA content using a NucleoCounter NC-3000 cell counter (ChemoMetec, Allerod, Denmark) according to the manufacturer’s instructions.

### Western blotting

Primary antibodies are listed in Supplementary Figure S7b. Cell extracts were made by lysis into SDS electrophoresis sample buffer. Western blotting was carried out as previously described (Dayal et al., 2009).

### Real-time PCR

RNA was extracted using RNeasy columns and real-time PCR were performed as previously described (Dayal et al., 2009) using *MARCH4* probe/primer set Hs00863129_m1 (Thermo Fisher Scientific). TBP was used for normalization.

### Data availability statement

The authors confirm that the data supporting the findings of this study are available within the article and its Supplementary Materials.

## ORCIDs

Angela McHugh: https://orcid.org/0000-0003-3020-4701

Kenneth Fernandes: https://orcid.org/0000-0002-9406-267X

Nerime Chinner: https://orcid.org/0000-0001-5891-7573

Adel F.M. Ibrahim: https://orcid.org/0000-0002-1066-7111

Amit K. Garg: https://orcid.org/0000-0001-7486-9284

Garry Boag: https://orcid.org/0000-0002-4656-2653

Lydia A. Hepburn: https://orcid.org/0000-0003-4851-6658

Charlotte M. Proby: https://orcid.org/0000-0002-3292-4836

Irene M. Leigh: https://orcid.org/0000-0001-8536-6439

Mark K. Saville: https://orcid.org/0000-0001-8057-0641

## Conflict of Interest

The authors state no conflict of interest.

## Figures and Tables

**Supplementary Figure S5a:**
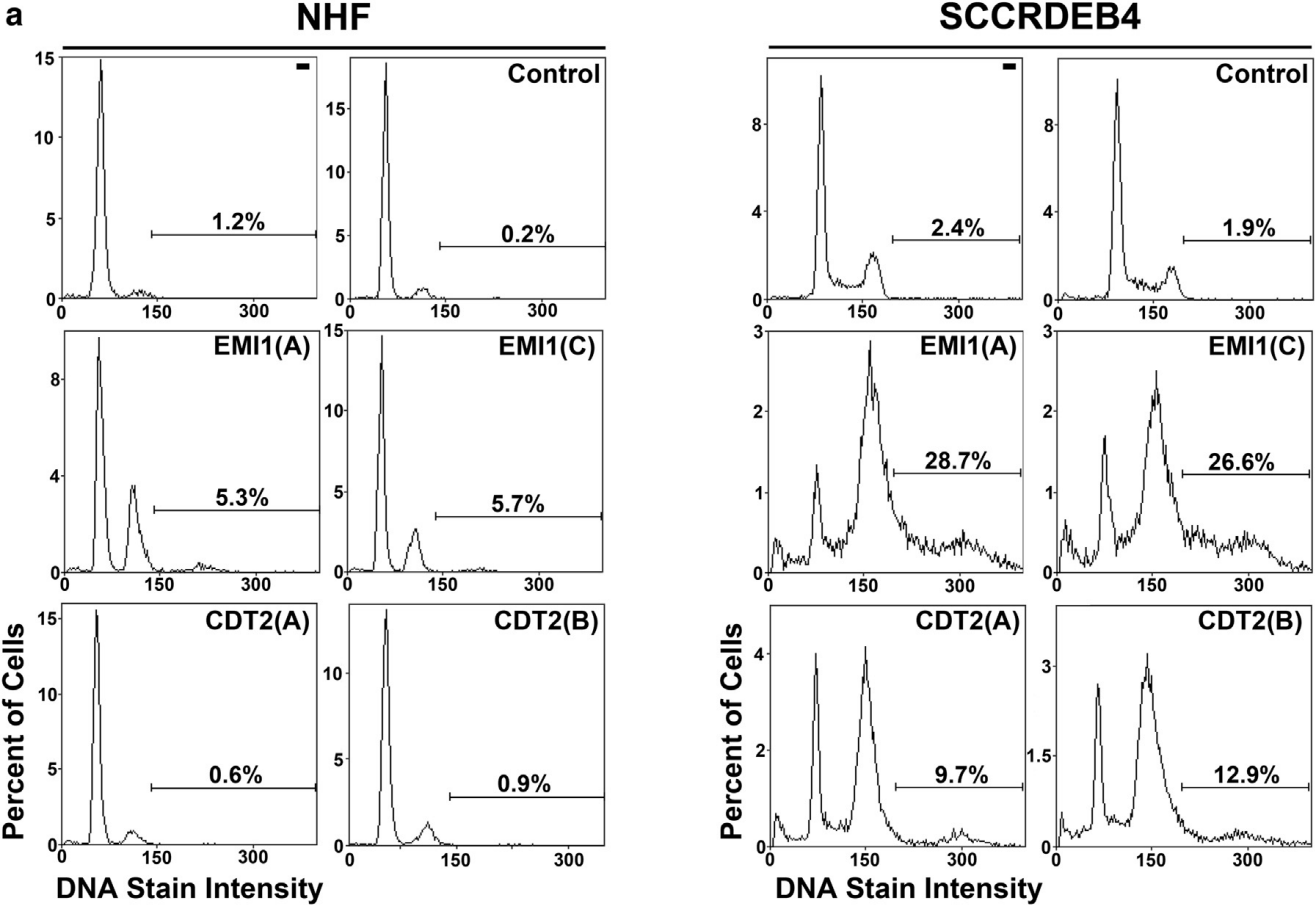
**EMI1 and CDT2 knockdown cause DNA re-replication in cSCC cells.** The DNA content of NHF and SCCRDEB4 cells was analyzed 72 hours after transfection with the indicated siRNA. The percentage of cells that underwent re-replication (>G2/M DNA content) is shown. cSCC, cutaneous squamous cell carcinoma; NHF, normal human fibroblast; siRNA, small interfering RNA.

**Supplementary Figure S5b:**
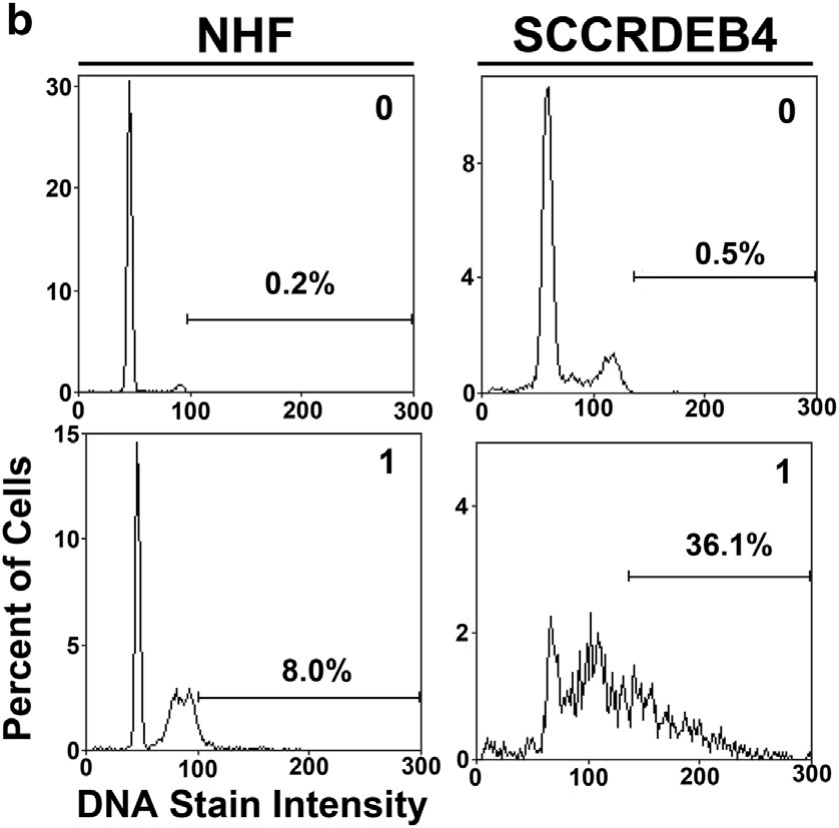
**MLN4924 promotes DNA re-replication in cSCC cells.** The DNA content of NHF and SCCRDEB4 cells was analyzed 48 hours after treatment with carrier (0) or 1μM MLN4924. The percentage of cells with >G2/M DNA content is indicated. cSCC, cutaneous squamous cell carcinoma; NHF, normal human fibroblast; siRNA, small interfering RNA.
